# No link between social admissions of elderly people and Christmas time

**DOI:** 10.1186/1757-7241-21-S2-A37

**Published:** 2013-09-09

**Authors:** Gitte Colaco, Thomas A Schmidt

**Affiliations:** 1 The Emergency Department, Holbaek University Hospital, Denmark

## Background

It is often said that Christmas and Public Holidays has an influence on people’s physical, social and emotional well-being, and Christmas is furthermore associated with traditions and family gatherings. But for some people Christmas does not bring joy or good memories; especially for the elderly with chronic diseases, bad family relations or no family at all, Christmas can be a troublesome time. Therefore it is almost mythically assumed that there is an increase in the number of social admissions of elderly during Christmas compared to similar dates not related to a Public Holiday. The scope of this study was to put this myth to the test.

## Methods

The year chosen for collection of data was the year 2011 and Christmas was defined to be on the 23rd, 24th and 25th December. Dates for comparison were chosen to be 11th, 12th and 13th November because November is a month that is closely related to December regarding seasonal diseases. The chosen weekdays are the same as in December. The study was performed as a chart review of patients referred to the General Department of Internal Medicine via the Emergency Department (ED):

### Inclusion criteria

Elderly aged >=70 years and an admission of maximum 24 hours (”unnecessary” admittance). The total number of admittance i.e. all ages and the admittance of patients >=70 years with a length of stay longer than 24 h were also captured.

## Results

**Table 1:** 

Admission	Total All ages	Total Age ≥ 70 years	24 hour admission
11-NOV-2011	44	16	5
12-NOV-2011	37	14	5
13-NOV-2011	42	16	2
Total November	123	46	12
23-DEC-2011	44	20	6
24-DEC-2011	36	11	4
25-DEC-2011	51	15	2
Total December	131	46	12

The reason for the admissions for both months was mostly related to complaints regarding pain. Most of the patients had a spouse or children, who they had contact with (Table 1).

## Conclusion

For short term admissions of elderly aged 70 years or more, the findings show, that there was no increase in social or any other admissions to the General Department of Internal Medicine via ED during the Christmas Holidays. A myth apparently discredited.

